# On Election to the Fellowship of the American Academy of Microbiology

**DOI:** 10.1128/mbio.00889-22

**Published:** 2022-05-11

**Authors:** Arturo Casadevall, Jennifer K. Lodge, Nguyen K. Nguyen

**Affiliations:** a Chair, Governors of the American Academy of Microbiology; b Chair, Subcommittee on Elections of the American Academy of Microbiology; c Director, American Academy of Microbiology

## EDITORIAL

Election to the American Academy of Microbiology (AAM) fellowship is among the greatest honors that a scientist working in the fields of microbial sciences can receive in their lifetime. In recent years, the AAM has pursued reform and renewal of the fellowship election, and this included formalizing the election process to make it more transparent, equitable, and inclusive. In this editorial, we detail the mechanics of the election process and provide advice to nominators and nominees in the form of frequently asked questions.

## THE FELLOWSHIP ELECTION PROCESSES

The election process begins in August with a call by the AAM for nominations from current AAM fellows. To be eligible to submit nominations, that fellow must be in good standing, which means that they must be in compliance with ASM and AAM paid membership dues. Each nomination for election must include one nominator and two supporters, each of whom must be good-standing AAM fellows. Criteria for election are provided on the AAM website (Criteria for Election to Fellowship in the American Academy of Microbiology [asm.org]), which also includes instructions for nomination and supporting forms. Starting in 2020, the AAM has promoted the process called “co-nomination” where potential candidates are highly encouraged to reach out to the current fellows to seek nominations. While the AAM does not allow candidates to apply directly, the co-nomination is a step forward to encourage candidates from the historically underrepresented communities to get nominated. Furthermore, the AAM now requests the nominees to share demographic information to allow the program to monitor our progress in increasing diversity among the fellows.

After the nomination deadline in the first week of October, the nominations are carefully inspected for completeness and eligibility by the AAM staff. The election process involves approval by vote from two independent bodies, the Sub-committee of Elections (SoE) and the Academy Governors (Governors), each consisting of AAM members elected by the entire membership of the Academy.

The nominations are first reviewed by the SoE, which usually meets in December. During the SoE review stage, each application is assigned to three reviewers based on the area of expertise, who independently vote “yes” or “no” to recommend the nominee for the AAM fellowship. Reviewers are asked to consider the impact that the nominee has had on their field beyond the traditional criteria of research and publication. There are five criteria that are scored: (i) professional accomplishments, (ii) publications, (iii) recognition and awards, (iv) service to microbial sciences, and (v) teaching and mentoring. They will grade each nominee in these five criteria and then decide if they will recommend each nominee by their “yes” or “no” vote. Reviewers have the flexibility to weigh these considerations when making a recommendation and are asked to limit their number of “yes” votes to 60% of their assigned nominees. Hence, each nominee must not only be qualified, but they must also be the top among the nominees to be recommended for the AAM fellowship. After the independent review, all nominees are brought to the whole SoE. Those with three “yes” votes are recommended to the Governors, and those with 2 “yes” and 1 “no” votes are deliberated intensively by the SoE. Though rarely, if a member of the SoE requests, nominees who received 1 “yes”/2 “no” votes have a chance to be deliberated by the SoE to ensure none of the nominees are overlooked. During the deliberation, the SoE reviewers provide short comments for their decision and vote on up to 85 nominees (65 recommended and 20 waitlisted) who will be referred to the Governors.

**FIG 1:**
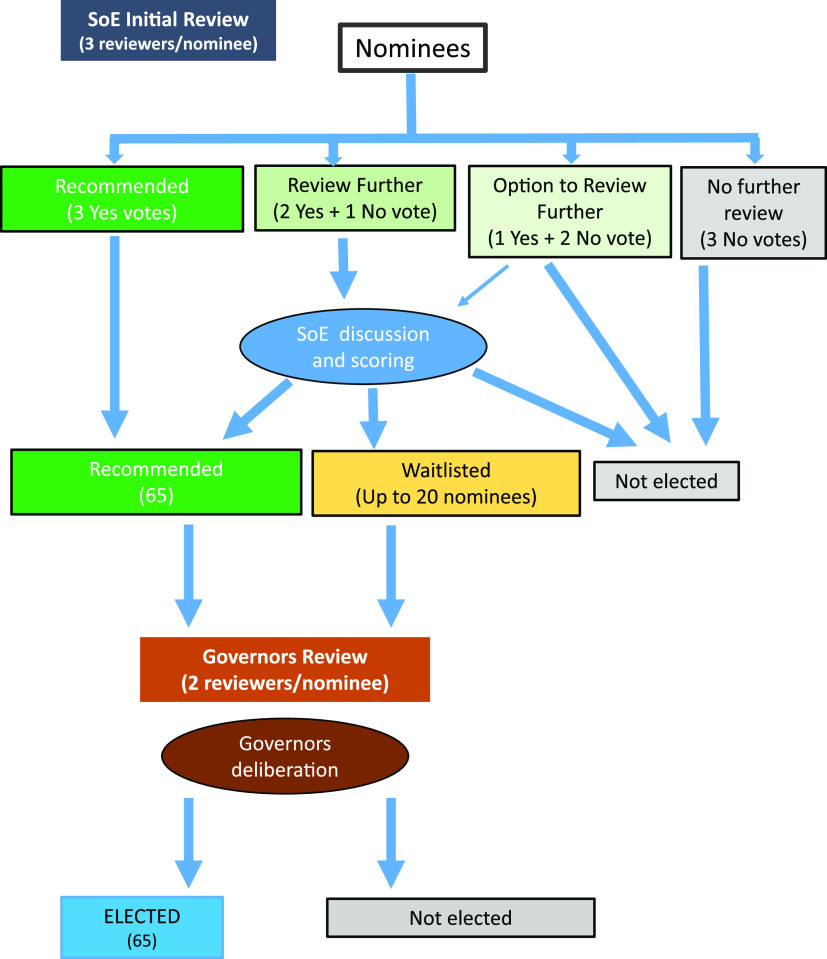
Schematic illustration of the AAM fellowship election review process.

The Academy Governors conduct the second round of review to take a deep dive on the 85 referred nominees. Each nominee is reviewed by two Governors in their area of expertise who aim to look at the nomination more holistically to decide if they agree with the SoE’s recommendation. Most of the time, the Governors concur with the SoE’s recommended nominees and take a closer look at the waitlisted. The Governors conduct the group’s final deliberation of the referred nominees in January. The nominees are discussed in the context of how their scientific qualifications compare to other nominees and whether their work promotes and advances microbial sciences and aligns with the vision of ASM and the Academy. After the deliberation, the Governors vote to elect the 65 nominees into the new class of Academy fellows. It is important to emphasize that each of the newly elected fellows is either supported by 5 reviewers in the Academy leadership or carefully vetted by the entire group(s). That makes the selection process very stringent but transparent and fair for all nominees. We note that other honorific societies such as the National Academies of Science, Engineering, and Medicine also set a limit on the yearly number of elected members. Having a limit forces careful consideration of nominees to ensure that the most meritorious are elected. ASM checks the Society’s ethics program records to determine whether there are any ethics violations or pending ethics complaints concerning any of the 65 newly elected fellows. Once this review is successfully completed for each individual, the election results for those new fellows are announced in mid-February via an ASM press release and different communication channels. The newly elected fellows are welcomed to the Academy and honored at a reception at the ASM Microbe Meeting in June (Fig. 1).

## SOME TIPS FOR SUCCESSFUL NOMINATIONS

The single most important criterion for election is the excellence of the contributions made by the nominee. However, with annual class slots being limited to 65 and many worthy applicants, the quality of the nomination package can make a difference in the outcome of the election. Here are some suggestions:

• Describe the nominee’s accomplishments in easily accessible language that makes the case for election. Although both the SoE and Governors are diverse groups composed of microbiologists with a wide range of expertise, one should not assume that excellence of the nominee contributions will be self-evident. Clearly articulate why the nominee should be elected to the AAM. Avoid jargon and excessive abbreviations. Do not assume that the reviewers will have specific expertise in the particular field of the nominee.
• Ensure that the set of publications listed in the application matches the stated accomplishments in content and scope. Sometimes there is a dissociation between the papers listed and the narrative of contribution. We suspect that some nominations list publications in high-impact journals rather than those that support the accomplishment narrative. This creates dissonance, can bewilder the reviewers, and does not help when there are multiple highly qualified nominees competing for limited slots. Election to the AAM is based on the excellence of contributions and not the impact factor of the journals listed for the publications listed.
• The AAM election process prefers individuals who have many contributions in diverse areas including scientific discovery, education, leadership, and service. Hence, the stronger nominations tend to include text describing a variety of efforts. In other words, state all the contributions and not just scientific discovery.
• Do not leave questions unanswered or sections blank.
• Read the nomination instructions on the AAM website carefully. Poorly prepared or incomplete applications are not likely to fare well in a very competitive process where the election slate is limited to 65.
• Start the nomination early and maintain a good communication between the nominator, supporters, and nominee to ensure the nomination materials are well coordinated and submitted on time. Late submission by one party can make a nomination ineligible for consideration.

## FREQUENTLY ASKED QUESTIONS

### 1. Is there a limit to the times that an individual can be nominated?

There is no limit on the number of times an individual can be nominated for fellowship. There is also no age limit for applicants. However, when a nominee is not elected, the nominator and supporters should consider whether the nomination can be significantly strengthened before resubmission. Here some judgement is important. Some nominations can be strengthened by better presentation while others require that the nominees make additional contributions to election. When an applicant is not elected, our advice is that nominator and supporters try to improve the strength of the application before it is resubmitted.

### 2. What does “waitlisted” mean?

It means that the nominee was referred to the Governors for further consideration. Given that most nominees are superb candidates, we use this term to reflect our hope that they will be elected in subsequent years. However, the waitlist is not kept once the election cycle concludes, and the waitlisted nominees are not announced. Hence, there is no waiting list, and for a waitlisted nominee to be considered again, a new application must be submitted to the AAM.

### 3. Does the name and reputation of nominators and supporters matter?

Of course, the name and reputation of those who nominate and second nominations matter, but not as much as some think. What really matters are the contributions of the nominee. Nominators and supporters in the field of the nominee who can clearly articulate the impact and excellence of the accomplishments are more important than choosing those with “big names” outside the area of expertise of the nominee.

### 4. Can I get feedback on how my nominee did during the election process?

The answer is no. The Academy Governors voted unanimously that no information be provided except for the outcome of the election. The reason for this is simple: the election process involves two distinct bodies, the SoE and Governors, who are involved in multiple deliberations. Thus, there is a high likelihood that any information provided would be incomplete and possibly inaccurate.

### 5. Should I infer anything from a failed nomination?

A failed nomination means that the nominee was ranked lower than others in the election process. Each year is different, with different compositions in the SoE, Academy Governors, and nominees mix. The election process is not an exact science, and every year there are judgment calls on who meets the criteria for the elected class. Hence, nominators, supporters, and nominees should not conclude that failure to get elected in one year means that nominee is not electable, just that the nomination did not receive sufficient support for election on that particular year. Moreover, as mentioned above, the presentation and information in the nomination package can shift the decision to one way or another. Many individuals require multiple nominations before they are elected.

### 6. Are international nominees at a disadvantage?

According to our data, the percentage of international nominees who have been elected to the Academy in recent years is on par with the nomination rate. Also, the Academy has a fair representation of international fellows, with 19% of the Academy fellows residing outside of the United States. Recently, the Academy has broadened the *Service* criterium to consider contributions to the field of microbiology at large, outside ASM and the U.S. This reflects the Academy’s efforts to promote diversity and inclusion in the membership and welcome excellence around the world.

### 7. How can I, a current AAM fellow, help strengthen the Academy fellowship?

All good-standing fellows are invited to identify and nominate excellent nominees to the Academy. The Academy especially welcome nominees from diverse backgrounds and from historically underrepresented communities. There are excellent microbiologists who are sometimes overlooked and not recognized by their peers. Fellows are encouraged to nominate these candidates. A more diverse and inclusive Academy is a stronger one.

### 8. What did the previous election process look like? Why were changes needed?

**FIG 2:**
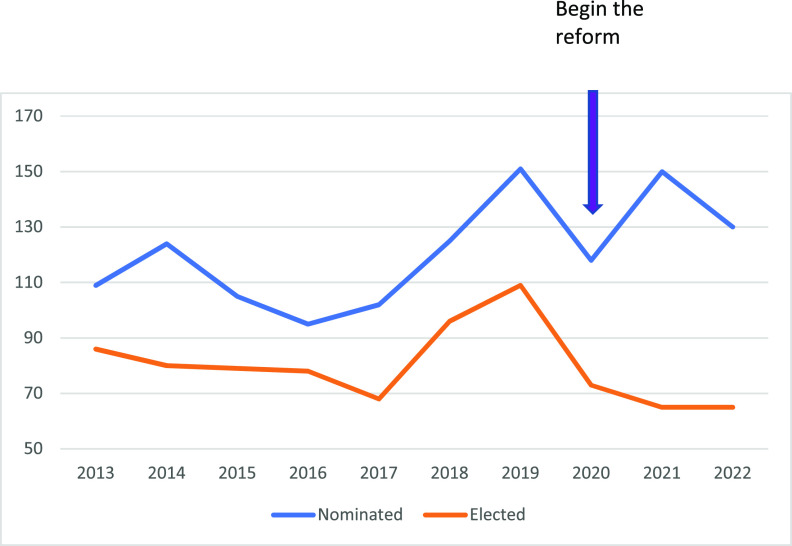
Number of AAM nominees (blue line) and elected Fellows (orange line) in the last 10 years.

The current election process is the result of a reform process starting in 2020. Prior to the reforms, there was year-to-year variability in the procedures used by both the Subcommittee on Elections and the Governors to select AAM fellows. Consequently, there was variation in the number of fellows elected each year (Fig. 2). Furthermore, it was not uncommon for the two review committees to disagree on the electability of some nominees, which was awkward and unpleasant. This led to the concern that without formal election protocols the unevenness in yearly results was haphazard and potentially unfair to nominees. To uphold the high standard of excellence and make the election process more consistent, fair, and transparent, the two committees set out to reform and codify the process of election, which is described above. One of the reforms was to set the yearly limit of newly elected fellows at 65, and this number was arrived at by analyzing the results of prior elections relative to number of nominees. Moreover, the 2022 election was the first year that self-reported gender and ethnicity data were collected on the nominees. This reform is a positive step toward building a diverse and inclusive Academy. The data in Table 1 suggest that nominees are elected in proportion to the demographics.

**TABLE 1:** Demographic information of nominees and elected fellows in the 2022 AAM election cycle. The nominees self-reported the data during the nomination process. Reporting data is voluntary, and individual demographic data were not disclosed to the reviewers nor used during the deliberation process

Self-declared category	Nominated		Elected	
	No.	%	No.	%
Gender				
Man	80	62%	35	54%
Woman	46	35%	26	40%
Gender not specified	4	3%	4	6%
Location				
U.S. based	105	81%	53	82%
International	25	19%	12	18%
Ethnicity of the U.S. nominees				
Asian	18	17%	7	13%
Black or African American	3	3%	3	6%
Hispanic, Latino, or Spanish	6	6%	3	6%
White	70	67%	36	68%
Other ethnicities	2	2%	2	4%
Multiracial	3	3%	1	2%
Prefer not to disclose	3	3%	1	2%

